# PurK, *N*^5^-Carboxyaminoimidazole Ribonucleotide Synthetase, an Exocrine Protein Induced by Potato Plants, Influences the Virulence Through Motility Modulation in *Pectobacterium brasiliense* NJAU180

**DOI:** 10.3390/microorganisms14030568

**Published:** 2026-03-02

**Authors:** Lingyan Xia, Yuanxu Zhuo, Nanqiao Lin, Na Yu, Shu Che, Chunting Wang, Liping Yang, Baishi Hu, Yanli Tian, Jiaqin Fan

**Affiliations:** 1 Laboratory of Bacteriology, Department of Plant Pathology, Nanjing Agricultural University, Nanjing 210095, China; 17798871122@163.com (L.X.); zhuoyuanxu187@163.com (Y.Z.); linnanqiao567@gmail.com (N.L.); yuna15288099526@163.com (N.Y.); shuche0523@163.com (S.C.); chuntingwct@163.com (C.W.); yangliping@yaas.org.cn (L.Y.); hbs@njau.edu.cn (B.H.); tianyanli@njau.edu.cn (Y.T.); 2 Jiangxi Provincial Key Laboratory of Agricultural Non-Point Source Pollution Control and Waste Comprehensive Utilization, Institute of Plant Protection, Jiangxi Academy of Agricultural Science, No. 602, Nanlian Road, Nanchang 330200, China; 3 Food Crops Research Institute, Yunnan Academy of Agricultural Sciences (YAAS), Kunming 650205, China

**Keywords:** *Pectobacterium brasiliense*, host-inducement, exocrine protein, motility, virulence, host-immune

## Abstract

Bacterial pathogens secrete effector proteins that suppress plant immune responses and facilitate infection. This study focuses on *Pectobacterium brasiliense* NJAU180, a bacterial pathogen causing severe blackleg disease in potato plants in Inner Mongolia, China. Using exoproteomic analysis, plant-induced extracellular proteins were identified by comparing culture supernatants from *P. brasiliense* NJAU180 grown in minimal medium (MM) alone and in the presence of aseptically grown potato plantlets at an early growth stage (OD_600_ ≈ 0.5). The results reveal PurK as a novel plant-induced extracellular protein, and deletion of *purK* markedly reduces virulence. PurK, *N*^5^-carboxyaminoimidazole ribonucleotide synthetase, is a key enzyme in *de novo* purine biosynthesis. Its impact on virulence is distinct from the conventional production of plant cell wall–degrading enzymes: PurK promotes motility by modulating transcription of flagellar genes, acting through its three domains as an integrated unit to infect successfully. Extracellularly detected PurK suppresses callose deposition, a PAMP-triggered immunity (PTI)-like defense, while it also triggers a strong hypersensitive response and upregulates expression of PTI marker genes such as *PR2* and *WRKY7* when secreted into the host plant. Although PurK interacts specifically with PurE, our data indicate that PurK’s pathogenic effects operate independently of purine biosynthesis. This study reveals a reliable experimental model for more accurate assessment of microbe–plant interactions and highlights new functional roles for PurK in *P. brasiliense* NJAU180 pathogenesis and identifies potential targets for disease control strategies.

## 1. Introduction

The strains from the genus of *Pectobacterium* are important plant pathogenic bacteria that infect various crops and cause severe soft rot disease. These bacteria produce a variety of enzymes that degrade cell walls, including pectinases, cellulases, and proteases. These enzymes disrupt the structure of the plant cell wall, particularly the middle lamella, resulting in tissue maceration and softening, and ultimately develop a viscous and unpleasantly odorous rot [1,2]. They have a broad host range, including many economically important crops from the Brassicaceae, Solanaceae, and Cucurbitaceae families [3]. These strains occasionally exist in a latent state within seemingly healthy plant organs, including potato tubers, taro corms, and the fruits of tomatoes and peppers [3,4]. Under conditions of high temperature and humidity during harvest, transportation, or storage, they proliferate quickly and spread widely, leading to extensive decay and substantial economic losses postharvest [5]. Bacteria–plant interaction is a dynamic and complex process of attack and defense. During infection, pathogens have evolved various virulence strategies to enhance their ability to colonize and infect host tissues. For instance, by forming biofilms either on the surface or within plant tissues, pathogens improve their adhesion, resist environmental stresses, and achieve persistent survival [6]. Some pathogens can produce phytotoxins that interfere with the host’s physiological metabolism and disrupt normal cellular functions [7].

In response to pathogen attacks, host plants have evolved a highly sophisticated innate immune system. The first line of defense is known as pathogen-associated molecular patterns (PAMP) triggered immunity (PTI). This system is activated when pattern recognition receptors located on the plant cell surface detect conserved molecular patterns associated with pathogens [8]. Early events in this immune response include a burst of reactive oxygen species and oscillations in cytosolic calcium concentrations [9], followed by the activation of mitogen-activated protein kinase (MAPK) signaling cascades. These pathways regulate the expression of defense-related genes and promote the synthesis of phytoalexins and pathogenesis-related proteins [10,11]. Simultaneously, plants reinforce physical barriers by depositing callose at the cell wall, effectively impeding further pathogen invasion [12].

Bacterial motility is one of the key virulence factors enabling the pathogens such as *Pectobacterium* to achieve successful infection [1]. This motility primarily relies on the flagellum—a highly conserved molecular machine that not only provides propulsive force but also participates in sensing environmental chemical signals, thereby regulating chemotactic behavior and guiding the bacterium toward specific host sites for targeted migration [13]. Structurally, the flagellum can be divided into three main parts: the basal body, the hook, and the filament. The basal body is embedded in the cell membrane and functions as a rotary motor driven by proton or sodium ion gradients. The filament, formed by the polymerization of flagellin, acts as a propeller, propelling the bacterium through liquid or semi-solid media [14,15,16]. The assembly of this sophisticated structure is under strict genetic regulation, involving multiple hierarchically expressed operons, with master regulators such as *flhDC* and *fliA* playing central roles in this hierarchical network [17]. Through flagellum-mediated motility, *P. carotovorum* can effectively traverse physical barriers on the plant surface, locate infection sites, and enhance its ability to migrate, colonize, and spread within host tissues, thereby increasing its pathogenic efficiency.

Purines are essential nitrogenous bases that constitute fundamental biomolecules such as DNA, RNA, and ATP [18]. In living organisms, the purine ring structure is primarily assembled stepwise via the *de novo* biosynthesis pathway [19]. A key enzyme in this pathway, PurK, catalyzes the fifth-step carboxylation reaction, converting the substrate CAIR (5-aminoimidazole-4-carboxamide ribonucleotide) into *N*^5^-CAIR. This step not only serves as a critical energy checkpoint in purine biosynthesis but also plays an important regulatory role in maintaining metabolic flux [20,21]. Researches have shown that mutations in the *pur* operon encoding PurK significantly attenuate bacterial virulence. For example, in *Brucella* species, the integrity of the purine biosynthesis pathway has been demonstrated to be essential for persistent infection in vivo, marking the first direct evidence linking purine synthesis and pathogenicity [22]. However, how PurK influences virulence remains unknown.

In this study, a secreted protein, PurK, was identified by comparing the exoproteome in the supernatant of pathogenic strain *P. brasiliense* NJAU180, which was grown in the presence and absence of aseptically grown potato plantlets, and a *purK* deletion mutant was created to systematically evaluate the impact of this gene on the growth and pathogenicity of NJAU180. The results demonstrate that PurK primarily influences virulence by modulating motility in NJAU180, unlike other strains of *Pectobacterium*, which rely on potent cell wall-degrading enzymes. It elucidates new functional roles of PurK in the pathogenesis of NJAU180 focusing on its secreted proteins.

## 2. Materials and Methods

### 2.1. Bacterial Strains and Plasmid Construction

The *purK* gene deletion mutant (Δ*purK*) and the domain-specific deletion mutants (Δ*purK*-N, Δ*purK*-M, and Δ*purK*-C) from the wild-type *P. brasiliense* strain NJAU180 were constructed using homologous recombination-mediated knockout technology. For functional complementation assays, the pBBR plasmid harboring the fragment of *purK* gene was introduced into Δ*purK* to generate the complemented strain Δ*purK* (*purK*). Similarly, pBBR plasmids carrying the respective domain fragments (*purK*-N, *purK*-M, or *purK*-C) were transformed respectively into Δ*purK*-N, Δ*purK*-M, and Δ*purK*-C to generate the corresponding complemented strains Δ*purK*-N (*purK*-N), Δ*purK*-M (*purK*-M), and Δ*purK*-C (*purK*-C). Meanwhile, the empty pBBR vector was transformed into Δ*purK* to obtain the control strain Δ*purK* (pBBR), which was used to assess the potential influence of the vector alone on subsequent phenotypic assays. All constructs were verified by PCR amplification and DNA sequencing.

### 2.2. Bacterial Strains, Plant Materials and Growth Conditions

The bacterial strains and plasmids used in this study are summarized in Supplementary Table S1. The wild-type strain *P. brasiliense* NJAU180 and its derivate strains were cultured in Luria-Bertani (LB) [23] or minimal medium (MM) [24] at 28 °C, and the derivate strains from *E. coli* were cultured in LB at 37 °C. The final antibiotic concentrations as shown below: kanamycin (Km) at 50 μg·mL^−1^, rifampin (Rif) at 100 μg·mL^−1^ and gentamicin (Gm) at 50 μg·mL^−1^. The optical density (OD) of the culture was measured by a BioPhotometer (Eppendorf, Hamburg, Germany) at 600 nm. Potato (*Solanum tuberosum* DM1-3) aseptic cultured seedlings were grown in 1/2 MS medium in an illumination incubator at 26 °C under a photoperiod at 16 h light/8 h dark. The plants of *Nicotiana benthamiana* were cultured in a greenhouse (temperature varying from 22 to 28, 16 h day length and an approximately 80% relative humidity).

### 2.3. RNA Isolation, cDNA Synthesis, qRT-PCR

Total RNA was extracted from *P. brasiliense* NJAU180 and its derived strains using an RNA extraction kit (Cat. No. AF505B; Proteinssci, Shanghai, China). To eliminate potential genomic DNA contamination, the extracted RNA was treated with DNase I. First-strand cDNA was then synthesized using Prime Script Reverse Transcriptase (Vazyme, Nanjing, China). Quantitative real-time PCR (qRT-PCR) was performed using the SYBR Prime-Script RT-PCR Kit (Cat. No. R323-01; Vazyme, Nanjing, China) on an ABI Prism 7500 Fast Real-Time PCR System (Applied Biosystems Inc., Foster City, CA, USA). The relative expression levels were calculated using the 2^−ΔΔCT^ method. All primers used in this study are listed in Supplemental Table S2, with the *recA* gene serving as an internal control as previously described [24].

### 2.4. Protein Extraction and Western Blotting

The wild-type strain *P. brasiliense* NJAU180 and its derived strains were complemented with a plasmid carrying the target gene fused with an epitope tagging fragment (DYKDDDDK) at the C-terminus as previously described [25]. Intracellular proteins were extracted using SDS lysis buffer (20 mM Tris-HCl, pH 7.0, 4% SDS, 20% glycerol), and extracellular proteins were collected as described previously [25,26]. The protein samples were separated by SDS-PAGE and transferred to a PVDF membrane for immunoblotting analysis. For detection of the FLAG-tagged target protein, the membrane was first incubated with a mouse anti-FLAG monoclonal primary antibody (1:3000 dilution; Cat. No. F1804, Merck KGaA, Darmstadt, Germany) at 4 °C overnight. After thorough washing, the membrane was then incubated with a horseradish peroxidase (HRP)-conjugated goat anti-mouse IgG secondary antibody (1:5000 dilution; Cat. No. 31430, Thermo Fisher Scientific Inc., Waltham, MA, USA) at room temperature for 1 h. Protein bands were finally visualized using an Ultra High Sensitivity ECL Kit (Cat. No. E412-01/02; Vazyme, Nanjing, China).

### 2.5. Extracellular Proteome Analysis

The wild type *P. brasiliense* NJAU180 and its derivative strains were co-cultured with 0.6 g aseptic, whole-plant potato grown plantlets in 20 mL MM medium, respectively. To avoid interference from intracellular proteins resulting from cell lysis, extracellular proteins were obtained from the supernatant of the strains after rapid interaction with the plantlets for 3 h at an initial concentration of OD_600_ = 0.1. Three independent biological replicates were prepared for each strain.

Protein samples were reduced, alkylated, and digested with Trypsin at a 1:50 (*w*/*w*) enzyme-to-substrate ratio. The resulting peptides were separated using an EASY nLC-1200 UHPLC system coupled online to a timsTOF Pro2 mass spectrometer. Following separation on a C18 column with an acetonitrile gradient, peptides were ionized at 1.5 kV and analyzed using the PASEF method [27]. The acquired MS/MS spectra were searched against the UniProt database using MaxQuant, with a false discovery rate (FDR) of <1% for peptide identification.

For label-free quantification, normalization based on total peptide intensity was performed to correct for sample loading variations and technical fluctuations. Specifically, peptide intensities in each sample were divided by the sum of all peptide intensities in that sample and multiplied by the average total intensity across all samples. Normalized intensities were used for subsequent statistical analysis.

To identify differentially expressed proteins (DEPs), a two-step screening strategy was employed. For initial screening, proteins with |fold change| > 1.2 and *p* < 0.05 were considered as potentially differential to ensure no candidates were missed. To obtain a more stringent list of candidates for downstream functional validation, the threshold was further raised to |fold change| > 2.0, combined with multiple testing correction.

Statistical analysis was performed using R software (version 4.2.1, R Foundation for Statistical Computing, Vienna, Austria) packages. Protein abundance differences between groups were compared using Student’s *t*-test. To control for multiple hypothesis testing, *p*-values were adjusted using the Benjamini–Hochberg method to control the false discovery rate (FDR). Proteins with |fold change| > 2.0 and adjusted *p*-value < 0.05 were defined as significantly differentially expressed.

Functional annotation of all identified proteins was performed using Gene Ontology (GO, http://geneontology.org/, accessed on 15 March 2025) and KEGG (http://www.genome.jp/kegg/, accessed on 15 March 2025) databases. Significantly, DEPs were further subjected to GO functional enrichment analysis and KEGG pathway enrichment analysis [28].

### 2.6. PurK Auxotroph Phenotype Validation Under Purine Supplementation

To evaluate the purine auxotrophy of the wild-type strain *P. brasiliense* NJAU180 and the *purK* mutant, the following procedure was employed. Both strains were initially cultured overnight at 28 °C in LB medium, then subcultured at a 1:100 ratio into fresh LB and grown to an OD_600_ of 1.0. Cells were harvested, washed, and resuspended in sterile water. Stock solutions of adenine, guanine, and hypoxanthine were prepared at a concentration of 3.46 g/L each. These stocks were incorporated into M9 minimal agar plates to achieve final purine concentrations of 27.68, 13.84, 6.92, 3.46, 1.73, and 0.86 mg/L, respectively. An M9 plate without any purine supplement served as the negative control. Resuspended cell suspensions were spotted onto the series of plates and incubated at 28 °C for 48 h, after which growth was visually assessed to compare the purine-dependent growth capacities of the two strains.

### 2.7. Pull-Down Analysis

Pull-down assays were performed as previously described [27]. Briefly, the His-purK expression plasmid was constructed by cloning the fragment of *purK* into the pET30a vector, while the fragment of *purE* was fused with a GST tag using the pGEX-6p plasmid. All recombinant plasmids were transformed into *E. coli* BL21 (DE3) competent cells. The transformed strains were cultured overnight at 37 °C, then diluted 1:100 into fresh LB medium and grown at 37 °C until the OD_600_ reached 0.4. Protein expression was induced with 0.1 mM isopropyl β-D-1-thiogalactopyranoside (IPTG) at 28 °C for 4 h. After centrifugation, the cell pellets were resuspended and lysed in 0.01 M PBS buffer (pH 7.4), and the supernatant was collected and concentrated. The concentrated tagged protein samples were incubated with 50 μL of GST affinity beads (GE Healthcare, Shanghai, China) in a final volume of 1 mL adjusted with 0.01 M PBS (pH 7.4) and incubated overnight at 16 °C. The beads were then washed six times with PBS buffer containing 1% Triton X-100 to remove non-specifically bound proteins. Finally, the bound proteins were eluted using glutathione elution buffer and analyzed by Western blotting. His-tag monoclonal antibody (M30111L) and GST-tag monoclonal antibody (M20007L) were used for detection.

### 2.8. Protein Structure Simulation

Three-dimensional homology models of full-length PurK and its N-terminal, M-terminal, and C-terminal domains were generated using the SWISS-MODEL server (https://swissmodel.expasy.org, accessed on 15 March 2025). The optimal model for each target sequence was selected based on the Global Model Quality Estimate score. Structural comparisons and visualizations of the modeled structures were performed using PyMOL software (version 2.5.0, Schrödinger, LLC, New York, NY, USA) [26].

### 2.9. Phylogenetic Tree Analysis

The amino acid sequences of homologous proteins from *P. brasiliense* NJAU180 and other representative strains, including *P. polaris*, *P. aquaticum*, *E. coli*, *Saccharolobus solfataricus*, *Brucella canis*, *Staphylococcus aureus* subsp. *aureus*, and *Acinetobacter baumannii*, were selected for phylogenetic analysis. Multiple sequence alignments were performed using ClustalX software (version 2.1, University College Dublin, Dublin, Ireland), and the resulting alignments were manually refined where necessary before being exported in FASTA format. The phylogenetic tree was constructed using the neighbor-joining method based on the neighbor-joining model with MEGA software (version 11.0, Pennsylvania State University, University Park, PA, USA). Visualization and layout optimization of the resulting phylogenetic tree were also conducted using MEGA software (version 11.0, Pennsylvania State University, University Park, PA, USA) [26].

### 2.10. Motility Assay

Swimming motility assays were performed as previously described [23]. Briefly, bacterial strains were cultured to logarithmic phase (OD_600_ = 1.0). A total of 2 μL aliquot of the bacterial suspension was spot-inoculated onto the center of MM plates containing 0.3% agar. After incubation at 28 °C for 18 h, the migration zone diameter was determined and photographed. For comparative purposes, all measured data were normalized to the mean of the control group.

### 2.11. The Activity of Plant Cell Wall Degrading Enzymes (PCWDEs) Assay

PCWDE activity of wild-type NJAU180 and its derivate strains was analyzed as previously described [25]. Bacterial cultures were grown to an OD_600_ of 1.0, spot-inoculated onto PCWDE activity assay plates independently, and incubated at 28 °C for 48 h. PCWDE production was evaluated by measuring the halos formed around the bacterial colonies.

### 2.12. Bacterial Two-Hybrid Analysis

The bacterial two-hybrid assay was performed following established methods [26]. The coding sequences of PurL, GuaA (NJAU180_1200), PurE, PurF, PurN, PurH, PurC, PurD, PurM, GuaA (NJAU180_1433), ThiC, and GuaA (NJAU180_1471) were cloned into the pTRG vector as previously described, while the fragment of *purK* was constructed into the pBT vector. The recombinant pTRG plasmids and *purK*-pBT were co-transformed into *E. coli* XL1-Blue competent cells. To validate protein–protein interactions, transformed cells were plated on selective medium containing 3-amino-1,2,4-triazole (3AT) and streptomycin. Protein interactions between PurK and each target protein were assessed based on bacterial growth.

### 2.13. Callose Deposition Assay

Callose deposition assays in *Nicotiana benthamiana* leaves were performed as previously described [23,29]. Wild-type strain *P. brasiliense* NJAU180 and its derivate strains were cultured to an OD_600_ of 1.2 and inoculated into leaves of 6-week-old greenhouse-grown *N. benthamiana* plants via syringe infiltration. After 18 h of incubation, the leaves were harvested and decolorized completely with 95% ethanol at 37 °C, followed by two washes with 70% ethanol and two washes with sterile water. The decolorized leaves were stained in 0.01% (*w*/*v*) aniline blue solution prepared in 150 mM K_2_HPO_4_ (pH 9.5) for 5 h in darkness. The stained samples were observed and imaged using an epifluorescence microscope (Olympus Corporation, Center Valley, PA, USA). Sterile water and *Pseudomonas syringae* pv. *tomato* DC3000 were used as controls.

### 2.14. Hypersensitive Response (HR) Assay

The hypersensitive response (HR) assay was performed as previously described [29]. Briefly, wild-type strain *P. brasiliense* NJAU180 and its derivate strains were cultured to an OD_600_ of 1.0, and the bacterial suspensions were infiltrated into the leaves of *N. benthamiana* using a sterile syringe. After inoculation, plants were maintained at 24 °C for 16 h, and the diameters of necrotic lesions were measured and photographed.

### 2.15. Agrobacterium-Mediated Transient Expression Assays

Agrobacterium-mediated transient expression assays were performed as previously described [30] with minor modification. Briefly, the fragment of target gene *purK* was cloned into the pBinGFP vector, and the resulting recombinant plasmid was transformed into *Agrobacterium tumefaciens* strain GV3101. After incubation, bacterial cells were harvested and washed twice with 10 mM MgCl_2_, then resuspended in infiltration buffer (10 mM MgCl_2_, 100 mM MES, 200 μM acetosyringone). The bacterial suspension was adjusted to an OD_600_ of 0.5 and incubated in the dark at 28 °C for 2 h without shaking. The prepared suspension was infiltrated into the leaves of *N. benthamiana* plants using a needleless syringe. Inoculated plants were maintained under 24 °C for 24 h before qRT PCR experimental analyses.

### 2.16. Flagella Staining

Flagellar staining was performed to observe the flagellar morphology of different strains. The tested strains were inoculated into LB liquid medium and cultured at 28 °C with shaking until the logarithmic growth phase. An appropriate volume of fresh bacterial culture was collected by centrifugation, and the cell pellets were washed twice with sterile water before being resuspended to a final concentration of approximately 10^8^ CFU/mL. A total of 10 μL aliquot of the bacterial suspension was applied to one end of a clean glass slide and air-dried naturally. The smear was then covered with flagellar staining solution and incubated at room temperature for 5–10 min. The staining solution was gently rinsed off with distilled water, and the slide was air-dried naturally. Flagellar morphology was observed under an oil immersion lens using an upright fluorescence microscope, and images were captured. At least 20 random fields were examined for each strain, and representative images were selected to illustrate flagellar characteristics.

### 2.17. Statistical Analysis

Each assay described above was repeated at least three times with 3–5 replicates in each.

Data on extracellular enzyme activities, pathogenicity, motility, and callose deposition are presented as the mean ± standard deviation (SD) (*n* = 3). Statistical analysis was performed using GraphPad Prism (version 9.0, GraphPad Software, Inc., San Diego, CA, USA) with one-way ANOVA followed by Dunnett’s post hoc test, with the wild-type strain NJAU180 serving as the control group, and differences were considered statistically significant at *p* < 0.05. Data from qRT-PCR and purine validation assays are presented as the mean ± standard deviation (SD) (*n* = 3). Statistical analysis was performed using GraphPad Prism (version 9.0, GraphPad Software, Inc., San Diego, CA, USA) with Student’s *t*-test (two-tailed). Data were confirmed to meet the assumptions of normality before analysis. Differences were considered statistically significant at *p* < 0.05.

## 3. Results

### 3.1. Inducement of Aseptic Grown Potato Plantlets on the Proteins Pectobacterium brasiliense NJAU180 Secreted Outside Membrane

Previous studies [31,32] have shown that pathogenic bacteria secrete many small molecule compounds into host plants in the course of infection. Our previous research demonstrated that plant extracts significantly induce the expression of virulence genes in *P. aroidearum* PccS1 [32].

In the present work, *P. brasiliense* NJAU180, isolated from potato soft rot samples in Inner Mongolia, China, was selected to analyze the exo-secreted proteins in the supernatant of NJAU180 cultured with aseptic grown potato plantlets.

Using a threshold of a fold change greater than 2.0 (i.e., log_2_FC > 1.0), compared to the wild-type strain cultured in minimal medium (MM), the supernatant of *P. brasiliense* NJAU180 cultured in MM supplemented with aseptic grown potato plants (NJAU180^plant^) showed a significant accumulation increase in 54 proteins and a decrease in 1268 proteins, indicating that the secretion of these proteins was markedly induced or suppressed during interaction with potato (Table S3).

To further focus on the exocrine proteins induced by the host, we implemented a stricter threshold of log_2_FC > 4.0. Under this criterion, compared to the sample of *P. brasiliense* NJAU180 cultured in MM only, the group from NJAU180^plant^ exhibited significantly enhanced secretion of 31 proteins and suppressed secretion of 694 proteins (Table S3).

### 3.2. PurK, a Potato Plant-Promoted Secretion Protein Conserved in Bacteria and Containing an ATP-Grasp Domain, Is Crucial to Pectobacterium brasiliense NJAU180 Virulence

Among the 31 plant-promoting exoproteins showing upregulated secretion (Table S3), there are seven proteins that exhibited significantly higher extracellular abundance compared to their counterparts in *P. brasiliense* NJAU180 cultures grown exclusively in MM. We selected 24 proteins which were detected only in the supernatant of NJAU180^plant^ rather than in that of NJAU180 cultured solely in MM; the later ones will be dealt with in the future works. Here, we selected these seven candidate proteins to investigate their potential roles in mediating the plant-enhanced exocrine protein effects on *P. brasiliense* NJAU180 virulence. Among the genes encoding these seven proteins, only deletion of *purK* resulted in a significant reduction in bacterial virulence, whereas deletion of any of the other genes did not affect the virulence of *P. brasiliense* NJAU180 (Figure S1).

Therefore, PurK was selected for further analysis to validate the proteomic findings and to establish a foundation for more detailed investigations into its pathogenicity. The 3 × FLAG tag, characterized by its small size and high hydrophilicity, minimizes interference with protein function, secretion, or localization while preserving native expression regulatory features, and has been widely used in bacterial studies [33]. In parallel, Western blot analysis of TCA-precipitated culture supernatants is a well-established technique for investigating protein secretion in bacterial systems [34,35,36]. Based on this, we complemented the strains with a plasmid carrying the purK gene fused to a C-terminal 3 × FLAG tag (DYKDDDDK) and employed the aforementioned method to assess the intra- and extracellular distribution of PurK.

**Figure 1:**
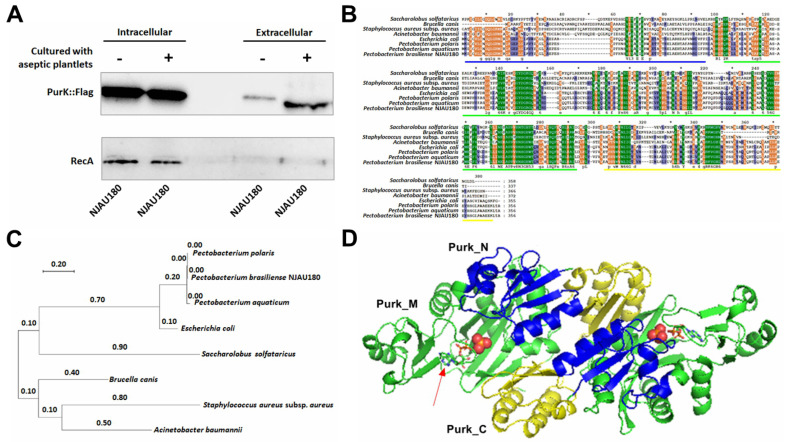
A highly conserved *Pectobacterium* protein, PurK, is promoted by interaction with the aseptic cultured potato plants to be secreted outside membrane in NJAU180. (**A**) PurK was blotted in the cells or supernatant of NJAU180 fused with an epitope fragment (DYKDDDDK) at the C-terminus of *purK*, and the bacterial cells were cultured in minimal medium (MM) in the presence and absence of aseptically cultured potato plantlets. RecA was used as the control. (**B**,**C**) Amino acid sequence alignments clustering and phylogenetic tree among *Pectobacterium brasiliense* NJAU180 and other bacterial strains. Sequence alignment of PurK proteins. Blue, green, and yellow underlines indicate the conserved N-termini, the conserved ATP-grasp domains, and the variable C-termini, respectively. These regions are discussed in the text. (**D**) Protein structure simulation of PurK. The red arrow indicates the ATP-grasp domain within each unit, which is responsible for ADP binding. The amino acid sequences of homologous proteins to be analyzed were sorted out, and the SWISS-MODEL was used for modelling analysis utilizing the comparative modelling method. The simulation results with the maximum GMQE value were selected, and Pymol software (version 2.5.0, Schrödinger, LLC, New York, NY, USA) was used for comparative analysis of the simulation results.

The results showed that PurK could be detected both inside and outside the wild type *P. brasiliense* NJAU180 cultured solely in MM, albeit a significantly lower proportion showed in extracellular. Interestingly, in the sample of NJAU180^plant^, there is a notable increase in extracellular accumulation of PurK, despite the intracellular levels remaining comparable to those observed in that of NJAU180 cultured in MM only (Figure 1A). These observations confirm that PurK is one of the exocrine proteins promoted by the plants.

Bioinformatic prediction using SignalP-5.0 revealed that PurK lacks a canonical N-terminal signal peptide (Sec/SPI probability = 0.0085; OTHER probability = 0.9913), suggesting it is not secreted via the classical Sec or Tat pathways. Consistent with this, SecretomeP 2.0 prediction for non-classical secretion yielded a low score (0.0001, below the threshold of 0.5), indicating that PurK does not conform to the defined model of constitutive non-classical secretion. However, Western blot analysis detected low but clear PurK signals in the culture supernatant under standard laboratory conditions (Figure 1A), contrasting with the bioinformatic predictions. This discrepancy suggests that PurK may be exported via an unconventional mechanism not captured by current prediction algorithms, which are primarily trained on constitutively secreted proteins. Notably, extracellular PurK abundance significantly increased upon co-culture with potato plantlets (Figure 1A), indicating that this unconventional secretion pathway is responsive to host-derived signals. These findings suggest that PurK may utilize a previously uncharacterized, host-inducible secretion mechanism that maintains basal activity even in the absence of host stimulation. Furthermore, it also reinforces the notion that plant-induced factors predominantly influence the secretion of PurK in *Pectobacterium* NJAU180.

PurK of *P. brasiliense* NJAU180 is a highly conserved protein, the deduced amino acid sequence exhibits 97–99% identity with those in the other two species of *Pectobacterium*, approximately 80% homology with that of *E. coli*, and lower with the others (Figure 1B,C). Among these proteins, the N-termini (highlighted with a blue underline) and ATP-grasp domains (indicated by a green underline) exhibit a high degree of conservation, while their C-termini (marked with a yellow underline) display significant variation (Figure 1B). Structural simulations suggest that PurK functions as a dimer, composed of two homodimers that band ADP through the ATP-grasp domain present in each unit (as indicated by the red arrow in Figure 1D).

### 3.3. The Gene purK Affects the Virulence by Influencing Motility Rather than Plant Cell Degrading Enzymes in Pectobacterium brasiliense NJAU180

**Figure 2:**
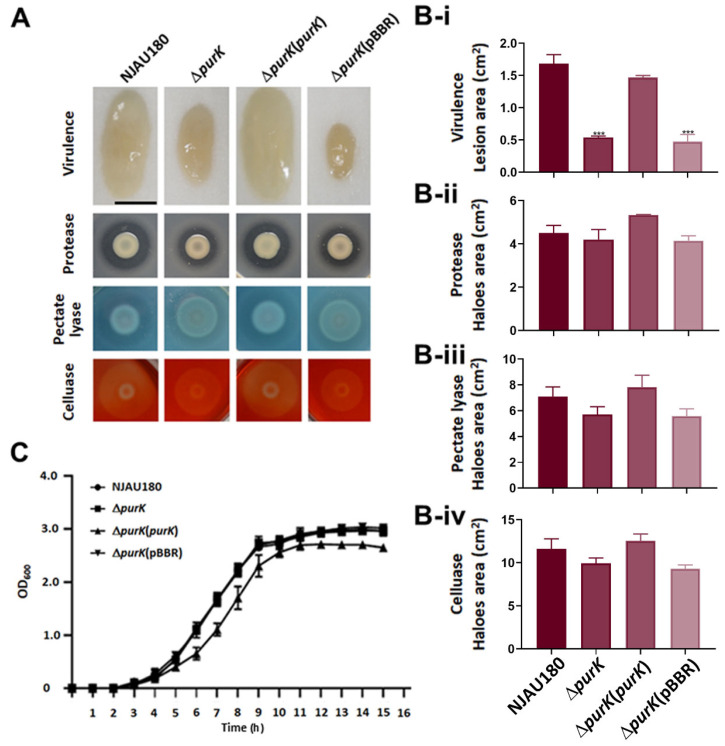
The virulence mediated by PurK operates independently of extracellular enzyme production. (**A**) Virulence assessment of NJAU180 and its derived strains, Δ*purK*, Δ*purK* (*purK*), and Δ*purK* (pBBR), on the leaves of Chinese cabbage (*Brassica rapa* subsp. *pekinensis*). Lesion sizes were measured 18 h after inoculation. Assays of extracellular enzyme activities (Pectinase, Cellulase, Protease) were visualized based on halos that occurred around the bacterial colonies when cells were spotted onto assay plates and incubated for 48 h at 28 °C. (**B**) Statistical analyses of the virulence assay (**B-i**) and extracellular enzyme activities assays (**B-ii**–**B-iv**). Bars represent relative maceration areas or halo areas of pectinase, protease, and cellulase activity caused by the indicated bacterial strains. All data are presented as the mean ± SD (*n* = 3). Statistical analysis was performed using one-way ANOVA followed by Dunnett’s post hoc test in GraphPad Prism 9.0, with the wild-type strain NJAU180 serving as the control group. *** *p* < 0.0001 versus the wild-type NJAU180; differences were considered statistically significant at *p* < 0.05. All data for different strains are presented in the same red color with varying opacity to ensure visual distinction while maintaining consistency: solid red for wild-type NJAU180, 75% opacity for Δ*purK*, 50% opacity for Δ*purK* (*purK*), and 25% opacity for Δ*purK* (pBBR). (**C**) Growth curves of the mutants and wild-type NJAU180 cultured in LB at 28 °C.

The virulence assay indicated that the maceration ability of the strain Δ*purK* was significantly lower than that of the wild type *P. brasiliense* NJAU180, and the decreased virulence of Δ*purK* could be restored by complementing the strain with the vector that carries *purK* fragment, rather with an empty vector (Figure 2A,B-i and Figure S1), though the multiplication of Δ*purK* in LB medium showed no difference with the wild type (Figure 2C), indicating that the gene *purK* is associated with virulence.

Plant cell wall degrading enzymes (PCWDEs) are widely recognized as the primary virulence factors in *Pectobacterium* [23]. However, the assays of PCWDEs activity showed that the derivate strains from NJAU180, including Δ*purK*, Δ*purK* (*purK*), and Δ*purK* (pBBR), exhibited similar levels of pectate lyase (Pel), celluase (Cel), and protease (Prt) activities compared to the wild-type NJAU180 (Figure 2A,B-ii–B-iv). The findings suggest that the virulence gene *purK* may employ a distinct mechanism in the modulation of virulence in *P. brasiliense* NJAU180.

**Figure 3:**
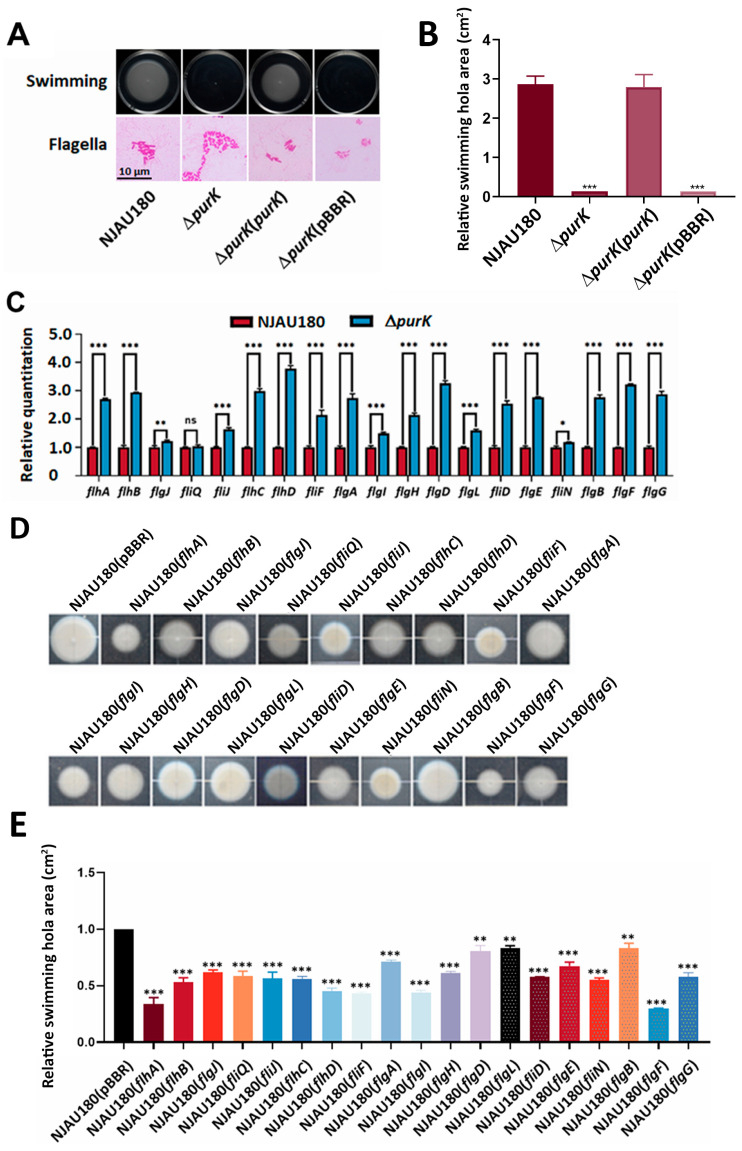
PurK negatively regulates flagellar gene expression. (**A**) Motility assay of NJAU180 and its derived strains, Δ*purK*, Δ*purK* (*purK*), and Δ*purK* (pBBR) on MM plates containing 0.3% agar. The diameters of the swimming halos were measured after 36 h at 28 °C. Images of the flagella from NJAU180 and its derived strains observed under microscope. Scale bars, 10 μm. (**B**) Bars present relative swimming halo size from three independent experiments. The swimming halo area of NJAU180 was set to 1.0. All data are presented as the mean ± SD (*n* = 3). Statistical analysis was performed using one-way ANOVA followed by Dunnett’s post hoc test in GraphPad Prism 9.0, with the wild-type strain NJAU180 serving as the control group. *** *p* < 0.001, (**C**) Relative mRNA expression of flagella-associated genes in NJAU180 and Δ*purK*. The mRNA level in NJAU180 was set to 1.0. The *recA* gene was used as an endogenous control. Data are presented as the mean ± SD (*n* = 3). Statistical analysis was performed using two-tailed Student’s *t*-test in GraphPad Prism 9.0. *** *p* < 0.001, ** *p* < 0.01, * *p* < 0.05, ns (not significant) versus NJAU180; differences were considered statistically significant at *p* < 0.05. (**D**) Motility assay of NJAU180 and its derived strains harboring plasmids for the overexpression of specific flagellar genes on MM plates containing 0.3% agar. The diameters of the swimming halos were measured after 36 h at 28 °C. (**E**) Bars present relative swimming halo size from three independent experiments. The swimming halo area of NJAU180 was set to 1.0. Data are presented as the mean ± SD (*n* = 3). Statistical analysis was performed using one-way ANOVA followed by Dunnett’s post hoc test in GraphPad Prism 9.0, with the wild-type strain NJAU180 serving as the control group. *** *p* < 0.0001, ** *p* < 0.001 versus NJAU180.

How does *purK* influence the virulence? Previous studies reveal that motility acts as an auxiliary virulence factor in many pathogenic bacteria [23,31]. Thus, the swimming motility of *P. brasiliense* NJAU180 and its derivates was assessed on MM containing 0.3% agar. The results demonstrated that Δ*purK* nearly completely lost its motility, and this ability can be restored in the Δ*purK* strain when complemented with a vector carrying the *purK* fragment, whereas the strain with the empty vector remained unchanged (Figure 3A,B).

The bacterial flagellum is a sophisticated organelle that plays a crucial role in bacterial movement, allowing them to swim actively towards host plants. Through chemotactic regulation, bacteria can navigate effectively, directing their movement towards nutrients and chemical signals released by the hosts. We first investigated flagellar morphology through staining, as flagellar assembly is essential for motility. Our observations revealed that the deletion of *purK* did not interfere with normal flagellation (Figure 3A). To understand how the *purK* gene influences bacterial motility, we measured the expression levels of 19 flagellar genes in both wild-type *P. brasiliense* NJAU180 and Δ*purK*. The results revealed that in Δ*purK*, expression of *fliQ* did not differ significantly from the wild-type NJAU180, whereas the other 18 genes were markedly upregulated, suggesting that *purK* acts as a negative regulator of flagellar gene expression at the transcriptional level (Figure 3C).

To assess the impact of these upregulated genes on bacterial motility, we overexpressed them in the wild-type *P. brasiliense* NJAU180 and analyzed their motility on the MM plates containing 0.3% agar. The results revealed that these strains overexpressing the *purK* gene significantly reduced bacterial swimming capacity (Figure 3D,E). This finding reveals a mechanism by which *purK* negatively regulates the expression of flagellar genes, thereby enhancing bacterial motility and promoting successful infection.

### 3.4. PurK Modulates the Virulence Through a Mechanism Operating Independently of Purine Biosynthesis

Purine is an essential precursor for the synthesis of key biomolecules such as nucleic acids and ATP in microbial cells. The *de novo* synthesis of purine nucleotides is a highly conserved multi-step metabolic pathway, in which the *purK* gene encodes phosphoribosylaminoimidazole carboxylase, an enzyme responsible for catalyzing the early rate-limiting step of converting CAIR to *N*^5^-CAIR, representing a critical node in the IMP biosynthesis pathway [19]. To elucidate the metabolic function of *purK* and its association with pathogenic phenotypes in NJAU180, this study systematically validated its role through exogenous purine supplementation experiments.

**Figure 4:**
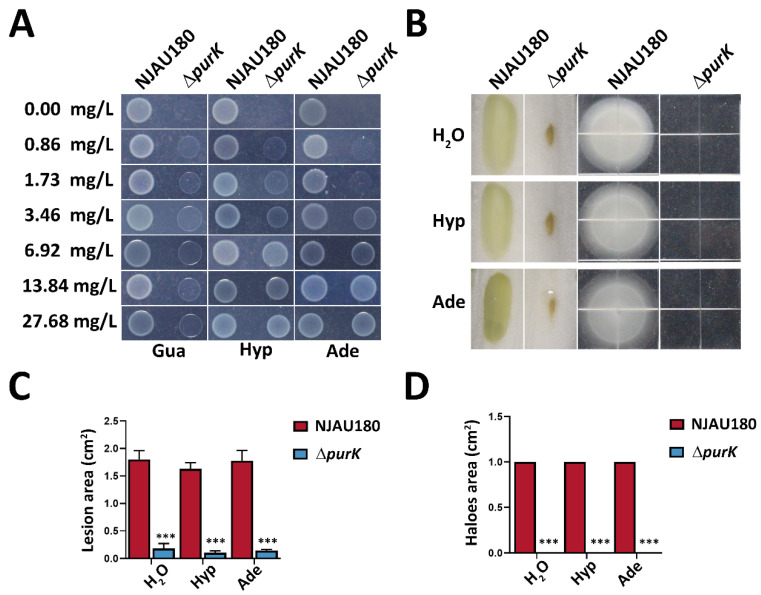
Exogenous purine restores growth but not virulence and motility in the *purK* mutant. (**A**) Purine auxotrophy validation of the *purK* mutant. Wild-type (NJAU180) and *purK* mutant strains were spotted onto M9 minimal agar plates supplemented with adenine (Ade), guanine (Gua), or hypoxanthine (Hyp) at the indicated concentrations (mg/L). Plates were incubated at 28 °C for 48 h. A control plate without purine (−) was included. The mutant failed to grow in the absence of exogenous purines. (**B**) Assessment of pathogenicity and motility restoration in the *purK* mutant by exogenous purines. The wild-type strain (NJAU180) and the *purK* mutant were cultured to OD_600_ = 1.0. Cells were resuspended in sterile water containing 13.84 mg/L adenine or hypoxanthine, with sterile water alone used as a control. For pathogenicity assessment, bacterial suspensions were inoculated onto leaves of Chinese cabbage (*Brassica rapa* subsp. *pekinensis*) and incubated at 28 °C. Lesion areas were measured 18 h post-inoculation. For motility analysis, the same suspensions were spotted onto minimal medium (MM) plates containing 0.3% agar and incubated at 28 °C for 36 h to observe the recovery of motility under purine supplementation. (**C**,**D**) Statistical analyses of the virulence assay (**C**) and motility assay (**D**). Bars represent relative maceration areas (**C**) or swimming halo areas (**D**) caused by the indicated bacterial strains. Data are presented as the mean ± SD (*n* = 3). Statistical analysis was performed using two-tailed Student’s *t*-test in GraphPad Prism 9.0. *** *p* < 0.0001 versus the wild-type NJAU180; differences were considered statistically significant at *p* < 0.05.

First, we assessed the growth capacity of the *purK* mutant in a chemically defined M9 minimal medium. Compared to the wild-type strain, the *purK* mutant completely failed to grow on M9 agar plates without exogenous purine supplementation, indicating a severe disruption in its purine synthesis pathway, consistent with a typical purine auxotrophic phenotype. To further dissect the specific step of this metabolic defect, we prepared a series of solid agar plates supplemented with varying concentrations of adenine (Ade), guanine (Gua), or hypoxanthine (Hyp). The results demonstrated that the *purK* mutant could not restore growth at any concentration of guanine, whereas on plates containing higher concentrations (13.84 mg/L and 27.68 mg/L) of either adenine or hypoxanthine, the mutant regained growth comparable to that of the wild type *P. brasiliense* NJAU180 (Figure 4A). These findings clearly indicate that the absence of *purK* disrupts IMP synthesis, leading to a complete loss of *de novo* purine synthesis capability in the strain, thereby confirming the indispensable role of *purK* in purine metabolism in NJAU180.

Having established the metabolic function of *purK*, we further investigated whether its influence on bacterial pathogenicity is mediated through the provision of purine precursors. The *purK* mutant was treated with 13.84 mg/L adenine and hypoxanthine, restoring its basic metabolic capacity to a level equivalent to that of the wild type, and its virulence and motility were subsequently evaluated. The results showed that although exogenous purine supplementation fully rescued the growth defect of the *purK* mutant on M9 medium, it did not restore its pathogenic capability toward the host or its motility (Figure 4B). This finding suggests that the role of *purK* in the pathogenicity of NJAU180 extends beyond merely supplying purine precursors for bacterial proliferation. Its contribution to virulence and motility appears to be independent of its metabolic synthesis function.

**Figure 5:**
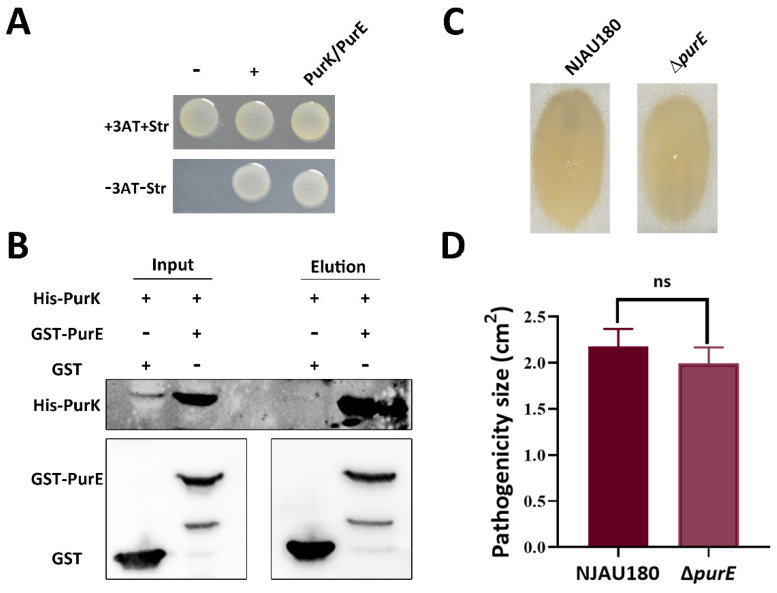
PurK interacts with PurE, yet PurE is dispensable for virulence in NJAU180. (**A**) Direct physical interaction between PurK and PurE was detected in *E. coli* using bacterial two-hybrid assay. Positive control (+), cotransformant containing pBX-R2031 and pTRG-R3133; negative control (−): cotransformant containing pBXcmT and empty pTRG. (**B**) Pull-down assay. Purified His-tagged PurK was immobilized on agarose beads and incubated with purified GST-tagged PurE or GST alone (control). After extensive washing, bound proteins were eluted and analyzed by immunoblotting using anti-GST and anti-His antibodies. (**C**) Virulence assessment of NJAU180 and Δ*purE* on the leaves of Chinese cabbage (*Brassica rapa* subsp. *pekinensis*). Lesion sizes were measured 18 h after inoculation. (**D**) Statistical analysis of the virulence assay. Bars represent relative maceration areas caused by the indicated strains. Data are presented as the mean ± SD (*n* = 3). Statistical analysis was performed using two-tailed Student’s *t*-test in GraphPad Prism 9.0. ns, not significant; differences were considered statistically significant at *p* < 0.05.

Studies have demonstrated that *purK* and *purE* are two functionally synergistic key genes in the bacterial purine biosynthesis pathway [37]. They jointly encode an enzyme system that catalyzes the same critical step: the carboxylation of AIR (5-aminoimidazole ribonucleotide) to CAIR (5-aminoimidazole-4-carboxylate ribonucleotide) [37]. To determine whether *purK* and *purE* have analogous roles during *P. brasiliense* NJAU80 infection, we initially carried out bacterial two-hybrid and pull-down assays. The results confirmed that PurK specifically interacts with PurE (Figure 5A,B).

To investigate the functional relationship between *purK* and *purE* in pathogenicity, a *purE* gene knockout mutant was constructed for virulence assay; the results showed that D*purE* exhibited no significant difference in virulence compared to the wild type (Figure 5C,D), indicating that *purE* is not essential for virulence in *P. brasiliense* NJAU180. This aligns with previous observations that the loss of pathogenicity in Δ*purE* likely arises from an independent role of PurK itself, rather than from its canonical function in purine biosynthesis.

The phenomenon of a metabolic enzyme possessing additional, non-metabolic functions is well established in bacteriology. Pancholi and Fischetti [38] first demonstrated that the glycolytic enzyme GAPDH functions as a surface adhesin and virulence factor in *Streptococcus*. Subsequent reviews have shown that such “moonlighting proteins”—including GAPDH, enolase, and chaperonins—are employed by over 90 bacterial species as virulence determinants [39,40]. Our finding that PurK plays dual roles in purine metabolism and virulence regulation, with its extracellular accumulation induced by plants, is consistent with the emerging paradigm of moonlighting proteins, raising the possibility that PurK may function as a novel moonlighting protein in *P. brasiliense* NJAU180.

### 3.5. Every Domain of PurK Is Indispensable for Virulence and Motility

**Figure 6:**
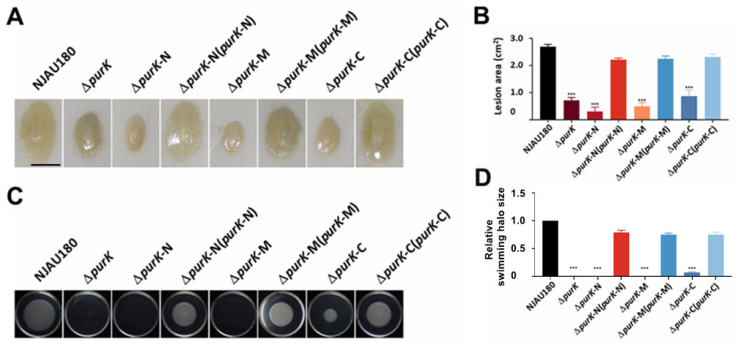
Roles of PurK domains in regulating virulence and motility. (**A**) Pathogenicity of the NJAU180, Δ*purK*, and strains with mutation in the fragment of PurK domain mutants (Δ*purK*-N, Δ*purK*-M, Δ*purK*-C), and the genetic complementation strains, Δ*purK*-N (*purK*-N), Δ*purK*-M (*purK*-M), Δ*purK*-C (*purK*-C), was evaluated on the leaves of Chinese cabbage (*Brassica rapa* subsp. *pekinensis*). (**B**) Statistical analysis of the virulence assay. Bars represent relative maceration areas caused by the indicated strains. Data are presented as the mean ± SD (*n* = 3). Statistical analysis was performed using one-way ANOVA followed by Dunnett’s post hoc test in GraphPad Prism 9.0, with the wild-type strain NJAU180 serving as the control group. *** *p* < 0.001 versus NJAU180; differences were considered statistically significant at *p* < 0.05. (**C**) Motility assay of these strains was carried out on MM plates containing 0.3% agar. The diameters of the swimming halos were measured after 36 h at 28 °C. (**D**) Statistical analysis of the motility assay. Bars represent relative swimming halo size from three independent experiments. The swimming halo area of NJAU180 was set to 1.0. Data are presented as the mean ± SD (*n* = 3). Statistical analysis was performed using one-way ANOVA followed by Dunnett’s post hoc test in GraphPad Prism 9.0, with the wild-type strain NJAU180 serving as the control group. *** *p* < 0.001 versus NJAU180; differences were considered statistically significant at *p* < 0.05.

Our structural simulation results suggest that PurK functions as a dimer, comprising two homodimer units. These units interact with ADP through the ATP-grasp domain located at the center. Notably, each unit possesses a conserved N-terminus and a variable C-terminus. (Figure 1B,C). To identify the key regions responsible for pathogenicity and motility, we constructed single domain knockout mutants targeting the C-terminal (*purK*-C), N-terminal (*purK*-N), and ATP-grasp (*purK*-M) regions, respectively. The virulence assays showed that removing any domain significantly reduced the virulence of *P. brasiliense* NJAU180, while complementation with the corresponding fragments restored the maceration ability and motility of the strains (Figure 6A,B). This functional recovery indicates that each of the constructed domains is properly and stably expressed [41,42,43]. However, compared with the other two mutants, the strain with a mutation in the C-terminal domain fragment exhibited a lesser reduction in virulence (Figure 6A,B).

Meanwhile, the motility assays revealed that the deletion of *purK*-C reduced bacterial motility, and the strains of both *purK*-N and *purK*-M impaired completely abolished motility, and the defects of motility were fully restored upon genetic complementation with the corresponding domains (Figure 6C,D). These results demonstrate that the structural integrity of *purK* is essential for the full pathogenicity and motility of *P. brasiliense* NJAU180. The N-terminal, C-terminal, and ATP-grasp domains function synergistically as an integrated functional unit in regulating virulence and motility, rather than serving redundant roles. Notably, the N-terminal and ATP-grasp domains are crucial in these processes. Their loss results in more pronounced phenotypic defects, suggesting that they serve essential functions in critical biochemical processes, such as purine metabolism or energy supply.

### 3.6. Extracellular PurK Suppresses Plant PTI Responses

**Figure 7:**
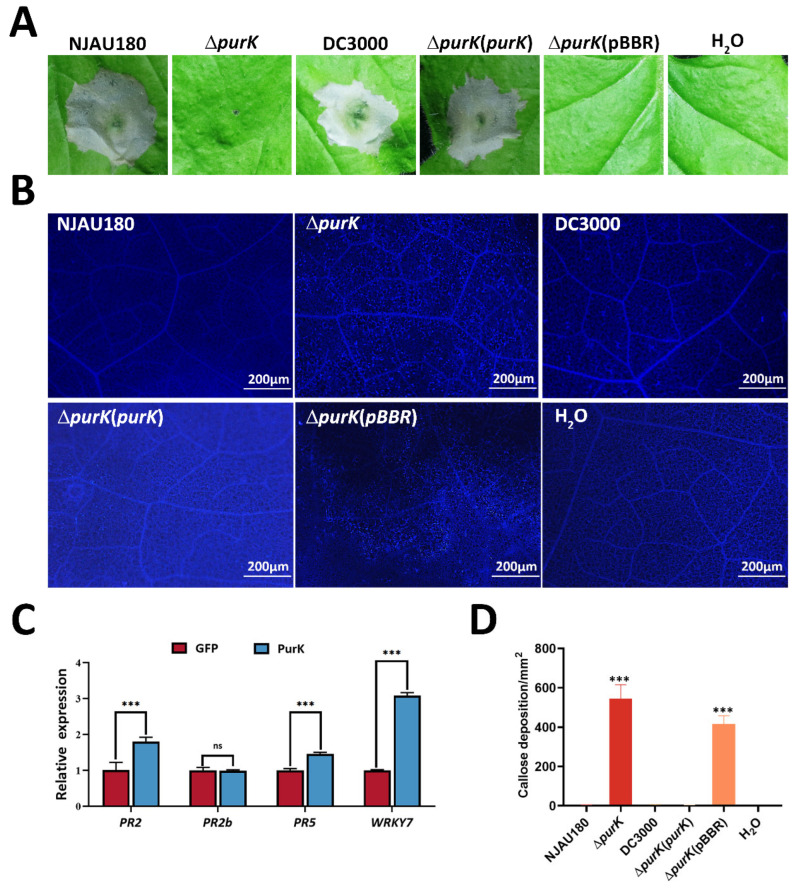
Extracellular PurK suppresses plant PTI responses. (**A**) Representative photographs of tobacco (*Nicotiana tabacum* cv. Xanthi) leaves at 24 h post-inoculation with NJAU180 and its derived strains, Δ*purK*, Δ*purK* (*purK*), and Δ*purK* (pBBR). Bacterial suspensions were infiltrated at 10^8^ CFU·mL^−1^. *P. syringae* pv. *tomato* DC3000 and sterilized water as controls. (**B**) Callose deposition in the *N. benthamiana* leaves infiltrated with NJAU180 and its derived strains. Portions of the *N. benthamiana* leaves were stained with aniline blue for callose deposition at 12 h after infiltration. *P. syringae* pv. *tomato* DC3000 and sterilized water as controls. The experiments were repeated three times with three internal replicates each. (**C**) Bars present mRNA relative expression of PTI downstream marker genes (*PR2*, *PR2b*, *PR5*, and *WRKY7*) in agroinfiltration with *Agrobacterium tumefaciens* carrying either an empty vector or a vector carrying the fragment of *purK*. The housekeeping gene *EF1α* was used as an endogenous control. Data are presented as the mean ± SD (*n* = 3). Statistical analysis was performed using two-tailed Student’s *t*-test in GraphPad Prism 9.0. *** *p* < 0.001, versus empty vector; differences were considered statistically significant at *p* < 0.05, ns indicates no significant difference. (**D**) Quantitative analysis of callose deposits. Data represent the mean number of callose deposits per mm^2^ (±SD) from three independent experiments (*n* = 3). Statistical analysis was performed using one-way ANOVA followed by Dunnett’s post hoc test in GraphPad Prism 9.0, with the wild-type strain NJAU180 serving as the control group. *** *p* < 0.001 versus NJAU180; differences were considered statistically significant at *p* < 0.05.

Previous studies have shown that pathogenic bacteria secrete effectors into host cells to target and suppress plant basal immunity, known as PTI, facilitating their infection and colonization of the plant [1,8,31]. In response, plants have evolved a multi-layered immune system to recognize pathogenic signals and activate defense mechanisms, including PTI mediated by pattern recognition receptors, as well as effector-triggered immunity (ETI), mediated by intracellular resistance proteins. To explore the role of the host-induced exocrine protein PurK in enhancing plant immunity, the observations of hypersensitive response (HR) and callose deposition were carried out with the wild type *P. brasiliense* NJAU180 and its derived strains, the results showed that Δ*purK* failed to elicit HR, but induced significant callose deposition in the *N. benthamiana* leaves (Figure 7A,B). The phenotypes of Δ*purK* (*purK*) closely resembled those of the wild-type, suggesting that PurK is essential for inhibiting callose deposition and triggering HR (Figure 7B). To confirm whether PurK activates PTI responses, we transiently expressed PurK in tobacco leaves and detected the transcript levels of PTI downstream marker genes (*PR2*, *PR2b*, *PR5*, and *WRKY7*) 18 h after inoculation. The results of qRT-PCR demonstrated that the expression of these genes was significantly upregulated (Figure 7C), suggesting that PurK strongly induces PTI-associated immune responses.

## 4. Discussion

PurK, A Plant-Induced Extracellular Protein, Suppresses Plant Immune Responses and Enhances Virulence by Promoting Motility with Its Three Domains

In this study, the supernatant of *P. brasiliense* NJAU180 co-cultured with aseptically grown potato plantlets in MM (NJAU180^plant^) was collected during the early growth stage for exoproteomic analysis. These profiles were subsequently compared with those derived from NJAU180 cultures grown solely in MM. The results of these analyses indicated that the supplementation of host plants in the medium significantly enhanced both the quality (Figure 1A) and diversity (Table S3) of exocrine proteins. It was found that only the strain without the fragment of *purK* significantly lost the maceration ability (Figure S1). In the previous study, PurK, *N*^5^-carboxyaminoimidazole ribonucleotide synthetase, has been demonstrated to play a crucial role in the *de novo* purine biosynthesis pathway [37,44], and it is a virulence-associated gene coding for factors involved in global bacterial physiology in *Yersinia pestis* [45]. Our findings reveal that PurK is a novel plant-induced secreted protein (Figure 1A). It suppresses host callose deposition while simultaneously triggering a hypersensitive response and upregulating the expression of plant immunity marker genes such as *PR2* and *WRKY7* (Figure 6). PurK contributes to the virulence of NJAU180 by enhancing bacterial motility (Figure 3A) modulated through its three domains as an integrated unit to infect successfully (Figure 5). These observations provide new insights into the pathogenic mechanism of *P. brasiliense* NJAU180 and establish an experimental system for studying microbe–plant interactions.

The Experimental Model of Pathogen Interaction with Aseptic Grown Plantlets Established in This Study Provides A Critical Framework for Analysing Bacterial Extracellular Proteins.

In natural environments, plants form complex symbiotic relationships with rhizospheric and phyllospheric microbiomes, which significantly influence pathogen infection processes and plant immune responses through mechanisms such as competition, antagonism, and immune modulation [46]. To investigate the key responses required for successful pathogen colonization under undisturbed conditions, in this study a simplified interaction system was established using aseptic grown potato plants (*Solanum tuberosum* DM1-3) co-cultured with the pathogen, and the exocrine proteins were collected at their early growth phase to avoid the influence of intracellular proteins resulting from pathogenic cell autolysis. This system not only effectively eliminates interference from environmental microbiota, ensuring that observed protein expression changes can be directly attributed to the interaction between pathogen *P. brasiliense* NJAU180 and the host as showed in the previous work [47], but also preserves the host’s tissue structure and fundamental physiological processes, thereby more accurately simulating natural infection scenarios—particularly suitable for focusing on molecular events during early infection stages [48].

Furthermore, we found that *P. brasiliense* NJAU180 exhibits slow growth in minimal medium (MM), and prolonged cultivation can easily induce cell autolysis. Concurrently, aseptic cultured seedlings may develop minor damage, both of which could introduce non-specific protein interference and compromise the accuracy of subsequent proteomic analyses. Therefore, this study focused on the early stage of pathogen–host interaction (3 h) to minimize background noise caused by plant tissue degradation and bacterial autolysis, ensuring that the detected extracellular protein profile authentically reflects biological processes related to infection [31], which is different from the inducement of plant-extract that was thought of as a nutrient [49], and avoids the contamination of the plant extracts from field products that might contain pesticides widely used in agriculture [50].

Inducement of Aseptic Grown Potato Plantlets on the Proteins *Pectobacterium brasiliense* NJAU180 Secreted outside Membrane/Outer Membrane Proteins as Pioneer Signals in Microbiota–Host Interactions.

Through exoproteomic analysis, this study revealed that during the early stages of pathogen infection, the host plant significantly suppresses the expression of multiple bacterial extracellular proteins. These suppressed proteins are primarily enriched in key pathways, including carbohydrate metabolism, amino acid metabolism, and membrane transport systems, as well as cofactor and vitamin metabolism (Table S3). These findings suggest that the host plant may actively interfere with the fundamental metabolism and transport capacity of the pathogen during early interaction, thereby limiting its pathogenicity.

Specifically, the host’s suppression of key enzymes involved in sugar and amino acid metabolism directly impairs the pathogen’s energy supply and biosynthesis of precursors, effectively disrupting its “metabolic engine”. This leads to energy deficiency and insufficient building blocks, hindering the synthesis of virulence-associated proteins and consequently inhibiting pathogen growth and reproduction [51]. Concurrently, the inhibition of membrane transport-related proteins severs the pathogen’s channels for nutrient uptake, toxin efflux, and signal communication, isolating it in a “metabolic island” within the host environment and impairing its ability to coordinate group behaviors and infection progression [52]. Furthermore, interference with cofactor and vitamin metabolism pathways affects multiple core biological processes, including energy metabolism, amino acid synthesis, and DNA replication, thereby establishing a multi-target defense network at the systemic level [53].

On the other hand, a total of 31 proteins were significantly upregulated under host induction, with functions mainly related to ABC transporters, purine synthesis, and partial carbohydrate metabolism pathways. This indicates that, in response to host-imposed nutritional immunity pressure, the pathogen enhances its active uptake of critical nutrients such as sugars, amino acids, and metal ions to sustain viability and virulence [54]. Notably, PurK, a key enzyme in the purine synthesis pathway, was markedly upregulated under host induction, suggesting its potential important role in the pathogen’s adaptation to the host environment and the establishment of a successful infection.

PurK Modulates the Flagellar Gene Expression to Control Motility for Successful Infection, rather than Stimulates PCWDEs in the Cells of *Pectobacterium brasiliense* NJAU180.

Previous studies have generally established that for soft rot pathogens such as *Pectobacterium* spp., extracellular enzymes (e.g., pectinases and cellulases) are key virulence factors, directly degrading plant cell wall structures and leading to tissue maceration and rot symptoms [55]. In this classical model, motility is often regarded as an accessory factor, primarily facilitating the later stages of pathogen spread and colonization within host tissues [56]. However, through systematic phenotypic analysis of the *purK* mutant, this study reveals a distinct pathogenic mechanism in *P. brasiliense* NJAU180. Extracellular enzymes are not the primary route through which *purK* regulates virulence. Instead, PurK significantly restrains bacterial motility by negatively regulating the expression of flagellar synthesis and related genes at the transcriptional level, and this impairment in motility is the key factor responsible for the marked reduction in pathogenicity. These findings suggest that, in NJAU180, efficient motility is not merely an accessory factor for colonization but may serve as an essential prerequisite for the pathogen to successfully locate and breach host physical barriers, thereby establishing initial infection.

Notably, the majority of flagellar genes were significantly upregulated at the transcriptional level in the Δ*purK* mutant, yet its swimming motility was markedly impaired. This apparent “transcription-up—phenotype-down” paradox suggests the existence of complex post-transcriptional or post-translational regulatory mechanisms intervening between flagellar gene expression and functional motility apparatus assembly. This seemingly paradoxical phenomenon has been previously documented. Dasgupta et al. [57] reported that inactivation of *fleN* in *Pseudomonas aeruginosa* resulted in upregulation of multiple flagellar promoters (e.g., *flgBCDE* by 27-fold, *fliLMNOPQ* by 5.6-fold) and loss of directional motility, although this was accompanied by hyper-flagellation (3–6 flagella per cell). In the present study, flagellar staining revealed no significant difference in flagellar number or morphology between the *purK* mutant and wild-type strains. This indicates that the motility defect in our mutant is not due to gross structural abnormalities in flagellar assembly, but rather to functional impairment of otherwise morphologically normal flagella—a finding that extends the observations of Dasgupta et al.

Based on these findings, we hypothesize that PurK may affect flagellar function through one or more of the following mechanisms: (i) altering translation efficiency or stability of flagellin proteins; (ii) impairing flagellar motor complex assembly or rotation; or (iii) disrupting chemotactic signaling. These potential mechanisms, which appear to operate independently of transcriptional regulation or flagellar assembly, warrant further investigation

The generality of this mechanism across other *Pectobacterium* strains or soft rot pathogens, and whether motility consistently contributes more significantly to virulence than extracellular enzymes in NJAU180, requires further investigation across broader genetic backgrounds and host environments.

PurK in *Pectobacterium brasiliense* NJAU180 May Function as An Immune Modulator when Present Extracellularly under Plant Inducement.

Traditionally, PurK has been widely recognized as a highly conserved metabolic enzyme dedicated to the *de novo* purine biosynthesis pathway. In model organisms such as *E. coli*, the *purK* gene is typically located within the *pur* operon and encodes an intracellular carboxylase that catalyzes an ATP-dependent carboxylation reaction, serving as a key component in the biosynthesis of inosine monophosphate (IMP). However, this study is the first to reveal a significant pathogenic role of PurK beyond its conventional metabolic function in a plant pathogenic bacterium.

Contrary to previous understanding, we found that PurK in *P. brasiliense* NJAU180 can be secreted extracellularly, a process significantly induced by plant signals, suggesting that it may possess biological activities independent of its metabolic role during pathogen–host interactions.

The detection of extracellular PurK under both basal and host-induced conditions, despite the absence of recognizable secretion signals, points to an unconventional export mechanism. The low SignalP-5.0 and SecretomeP 2.0 scores rule out classical Sec/Tat-dependent secretion and constitutive non-classical pathways as defined by current models [58]. Yet, the clear immunoblotting signals in culture supernatants suggest that PurK employs an export route that falls outside the training space of existing prediction algorithms. This host-responsive secretion pattern is reminiscent of stimulus-coupled export systems described in other bacterial pathogens, in which basal secretion maintains a ready-to-respond pool while host contact triggers rapid mobilization [59]. The identification of PurK as a protein that defies conventional prediction underscores the limitations of relying solely on bioinformatic tools for secretome analysis and highlights the value of empirical approaches, particularly under infection-mimicking conditions. Elucidating the precise mechanism—whether involving outer membrane vesicles, a dedicated secretion system, or other pathways—will require further investigation.

Furthermore, extracellular PurK was shown to play a dual role in plant immune regulation: on one hand, it suppresses plant PTI responses such as callose deposition, while on the other hand, it elicits a strong hypersensitive response (HR) in tobacco and significantly induces the expression of PTI marker genes. These findings imply that secreted PurK may function as a novel effector protein, participating in the complex network of plant immune signaling.

In summary, findings from this study raise the possibility that PurK could have dual functions in bacterial pathogenesis: beyond its canonical role in purine biosynthesis, it may function as a plant-induced secreted protein that influences bacterial motility and virulence through modulation of flagellar gene expression. Additionally, PurK exhibits biphasic immunomodulatory activity, suppressing early PTI responses while later triggering HR and defense gene upregulation. While these findings provide new insights into the complexity of bacterial pathogenicity, we acknowledge that several questions remain—including the precise secretion mechanism, the role of protein stability in domain function, and the plant recognition components involved. Future studies addressing these limitations will further elucidate the multifaceted roles of PurK in pathogen–host interactions. These observations also suggest that other conventional metabolic proteins may possess yet-unexplored non-canonical functions, providing new theoretical insights and potential targets for developing control strategies against plant bacterial diseases.

## 5. Conclusions

This study, through secretory proteomic analysis, revealed that *Pectobacterium brasiliense* NJAU180 secretes a plant-induced protein, PurK, during early interaction with its potato host. While PurK is a key enzyme in the purine biosynthesis pathway, we demonstrate that in *P. brasiliense* NJAU180, it retains its canonical enzymatic function and significantly influences bacterial virulence. Unlike traditional *Pectobacterium* that primarily rely on extracellular enzymes for pathogenicity, PurK promotes bacterial motility by modulating flagellar gene expression, functioning as an integrated unit through its three distinct domains. Furthermore, extracellularly detected PurK suppresses callose deposition in plants, simultaneously eliciting a hypersensitive response and upregulating the expression of PTI marker genes.

We acknowledge that several questions remain, including the precise secretion mechanism, the role of protein stability in domain function, and the plant recognition components involved. Future studies addressing these limitations will further elucidate the multifaceted roles of PurK in pathogen–host interactions. Nevertheless, this study systematically elucidates the multiple functions of PurK in the pathogenesis of NJAU180, offering novel potential molecular targets for developing disease control strategies against this pathogen.

## Data Availability

The data supporting the findings of this study are available from the corresponding author upon reasonable request for legitimate research purposes, subject to any applicable restrictions.

## Supplementary Materials

The following supporting information can be downloaded at: https://www.mdpi.com/article/10.3390/microorganisms14030568/s1, Figure S1: Virulence assays of strains with mutations in the genes encoding exocrine proteins that were differentially expressed in NJAU180 grown in minimal medium (MM) supplemented with aseptically cultured potato plantlets. Table S1: The strains and plasmids used in this study. Table S2: The primers used in this study. Table S3: The significantly differentially expressed proteins in the supernatant of NJAU180 cultured in MM supplemented with aseptic potato plants (showed as NJAU180 Plant) compared with that of NJAU180 cultured solely in MM.
