# Supplementary material for: PurK, N5-Carboxyaminoimidazole Ribonucleotide Synthetase, an Exocrine Protein Induced by Potato Plants, Influences the Virulence Through Motility Modulation in Pectobacterium brasiliense NJAU180
Source: Microorganisms. 2026 Mar 2;14(3):568. doi: 10.3390/microorganisms14030568 (PMC13028927; doi:10.3390/microorganisms14030568)
Supplement: Supplementary file 1 [file microorganisms-14-00568-s001.zip › Figure S1.pdf]

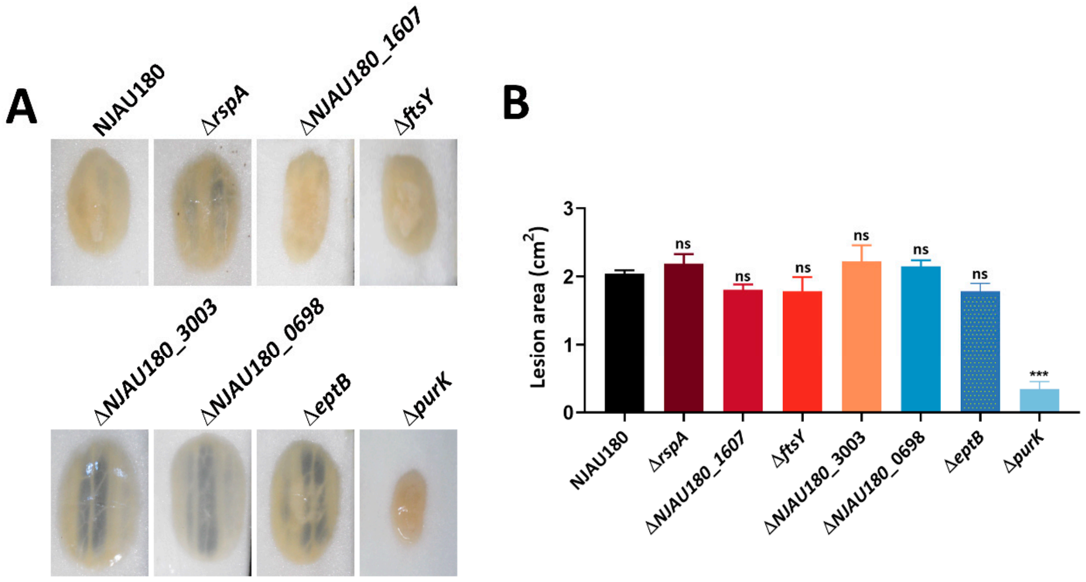

Figure S1. Virulence assays of strains with mutations in the genes encoding exocrine proteins that were differentially expressed in NJAU180 grown in minimal medium (MM) supplemented with aseptically cultured potato plantlets. A, Virulence assessment of NJAU180 and its derived strains, Δ*rspA*, Δ*NJAU180_1607*, Δ*ftsY*, Δ*NJAU180_3003*, Δ*NJAU180_0698*, Δ*eptB* and Δ*purK* on the detached leaves of Chinese cabbage (*Brassica rapa* subsp. *pekinensis*). Lesion sizes were measured 18 h after inoculation. B, Statistical analysis of the virulence assay. Bars represent the calculated relative maceration areas. Data are presented as the mean ± SD (n = 3). Statistical analysis was performed using one-way ANOVA followed by Dunnett's post-hoc test in GraphPad Prism 9.0, with the wild-type strain NJAU180 serving as the control group. ***$P <$ 0.001 versus NJAU180; differences were considered statistically significant at $P < 0.05$, *ns* indicates no significant difference.
